# Smartphone-Based Markerless Motion Capture for Spatiotemporal Gait Assessment: Applied Within-Session Reliability and Comparability of OpenCap Versus OptoGait

**DOI:** 10.3390/s26041234

**Published:** 2026-02-13

**Authors:** Christopher James Keating, Matteo Vitarelli, Domenico Cherubini

**Affiliations:** 1 Facultad de Deportes, Universidad Católica San Antonio de Murcia, 30107 Guadalupe, Spain; dcherubini@ucam.edu; 2 Department of Human Sciences and Promotion of the Quality of Life, San Raffaele Open University of Rome, 00166 Rome, Italy; matteo.vitarelli@univr.it; 3 Department of Neurosciences, Biomedicine and Movement, University of Verona, 37134 Verona, Italy

**Keywords:** OpenCap, OptoGait, gait, gait analysis, spatiotemporal, markerless motion capture, reliability, SEM, MDC

## Abstract

Objective gait assessment is increasingly needed beyond specialized laboratories, and 3D markerless motion capture is emerging as a viable option; however, evidence regarding its applied repeatability and practical use for spatiotemporal gait outcomes in scalable clinical and field settings remains limited. This study evaluated the applied repeatability and practical comparability of OpenCap (camera-based; CM) versus a commonly accepted photoelectric walkway (OptoGait; OPT). Thirty-nine healthy adults completed three 10-m overground trials at self-selected speed. CM parameters were derived from OpenCap’s Advanced Overground Gait Analysis. Within-device reliability was good-to-excellent for gait speed, stride length, and cadence (ICC (3,1) = 0.734–0.920 OPT; 0.791–0.917 CM) and excellent when averaging three trials (ICC (3,3) = 0.892–0.972 OPT; 0.919–0.971 CM); double support showed lower reliability (ICC (3,1) = 0.527 OPT; 0.647 CM). Between devices, CM showed higher mean speed (+0.110 m/s), stride length (+0.127 m), and double support (+3.17% of the gait cycle), while cadence was very similar (−0.59 spm). Correlations were high for speed (r = 0.951), stride length (r = 0.864), and cadence (r = 0.983) but moderate for double support (r = 0.405); absolute-agreement ICCs were highest for cadence (0.980) and lowest for double support (0.271). OpenCap provides reliable within-session estimates for key spatiotemporal measures, but systematic bias indicates it should be used consistently as a standalone tool rather than interchangeably with OptoGait without device-specific correction or reference values.

## 1. Introduction

Gait is a core functional behavior that integrates neuromuscular control, musculoskeletal capacity, balance, and cardiopulmonary reserve, and objective gait assessment is therefore widely used to characterize mobility limitations, stratify risk, and evaluate intervention response across clinical and community settings [1,2,3]. In particular, spatiotemporal gait parameters are practical, interpretable outputs that can be collected quickly and mapped to clinically relevant outcomes [1,2,3,4]. Among these outcomes, gait speed has been widely promoted as a global marker of functional health, often described as a “functional/sixth vital sign” because it is feasible to obtain, interpretable, and strongly associated with clinically relevant outcomes in diverse populations [3,5]. Although gait speed is commonly considered a functional indicator, it reflects the combination of cadence and step length. Consequently, similar gait speeds may result from very distinct spatiotemporal strategies [4,6,7]. Therefore, evaluating all gait parameters is essential for a more precise interpretation of mobility and functional changes in gait.

The clinical value of assessing gait is reinforced by longitudinal and pooled-cohort evidence linking slower gait patterns to higher risk of falls and mortality in older adults, supporting its role in prognosis and risk stratification [1,7]. In rehabilitation and intervention studies, spatiotemporal metrics are also commonly used as responsive outcomes; however, meaningful interpretation requires measurement systems with known precision and minimal detectable change, particularly when expected changes are small to moderate at best [8].

Despite its utility, comprehensive three-dimensional gait analysis has historically been constrained by the cost, space requirements, technical expertise, and time burden associated with laboratory-based marker-based motion capture and force platforms [2,9,10,11]. These barriers are especially consequential when the goal is to scale gait assessment beyond specialized laboratories and into routine clinical workflows, community sites, and field-based testing for at-risk or hard-to-reach populations, where portability, rapid setup, and standardized procedures become decisive factors [12]. To address these implementation constraints, instrumented walkways and optical or sensor-based timing systems have become established and validated options for efficiently quantifying spatiotemporal gait outcomes [13,14,15].

Within the broader ecosystem of clinical gait assessment tools, the Microgate OptoGait photoelectric cell system has become a well-established, practical approach for spatiotemporal gait measurement. OptoGait’s deployment advantages, portable hardware, rapid trial acquisition, and automated parameter extraction make it attractive for clinics, community programs, and research contexts where laboratory infrastructure is limited or participant burden must be minimized [14,16,17]. Its measurement performance has been supported by prior validation work against established walkway comparators, including studies reporting strong concurrent validity for several spatiotemporal parameters [14,16,17,18].

At the same time, three-dimensional markerless motion capture (3D MMC) has rapidly expanded due to advances in computer vision and pose estimation, enabling the extraction of gait measures from standard video without reflective markers or specialized camera arrays, thereby offering greater accessibility and scalability without complex setup [19,20]. Recent systematic reviews and meta-analyses generally support the good-to-excellent performance of markerless approaches for spatiotemporal gait parameters compared with marker-based references; however, they also highlight heterogeneity in camera configurations, calibration procedures, processing pipelines, and outcome definition factors that can meaningfully influence validity and reliability and complicate comparisons across studies [19,21,22,23]. This heterogeneity has motivated a growing focus on methodological transparency and protocol standardization, as well as targeted psychometric work (validity, test–retest reliability, and measurement error) in specific populations and deployment contexts [21,24].

OpenCap, in particular, is an open-source, smartphone-based 3D MMC platform developed to reduce traditional barriers to biomechanical measurement by enabling video capture on consumer iOS handheld devices and automated cloud-based processing [12]. The foundational OpenCap validation work emphasizes scalability and accessibility as primary design objectives, positioning the tool for large-scale studies and real-world deployments where conventional motion laboratories are impractical [12]. Further investigation has extended the evidence base by evaluating OpenCap in gait contexts that include non-typical patterns, further underscoring both its promise and the value of rigorous validation among tasks and populations [25].

To use markerless gait methods in clinical and field settings, it is essential to have standardized procedures for collecting data. These procedures help ensure results are repeatable and enable confident interpretation of changes, accounting for known measurement errors and minimal detectable change thresholds [21,26]. This need is directly addressed by our recently published standardized gait analysis protocol for 3D MMC, which provides explicit guidance on camera placement, calibration, and trial procedures, and reports reliability/precision benchmarks to support interpretation in applied settings [27]. Building on that protocol-driven foundation, a direct comparison of OpenCap-derived spatiotemporal outcomes with a well-established clinical reference tool, such as OptoGait, offers a practical way to evaluate and contrast the similarity and difference between an easily deployable markerless system (OpenCap) and a more equipment-intensive clinical platform (OptoGait), while establishing interpretable reliability and measurement error benchmarks for real-world gait assessment.

Therefore, the present study aims to evaluate the applied repeatability and practical comparability of spatiotemporal gait parameters obtained with 3D MMC (OpenCap) compared with an established photoelectric cell system (OptoGait) in young, healthy adults. By explicitly pairing a scalable markerless workflow with an established, clinically practical comparator system, this work aims to strengthen the evidence base for field- and clinic-oriented gait quantification using 3D MMC.

## 2. Materials and Methods

### 2.1. Participants

Thirty-nine healthy adults from the university (*N* = 39; 15 females, 24 males) voluntarily participated. Participants reported no known neurological, musculoskeletal, or cognitive impairments that could affect normal walking. Written informed consent was obtained in accordance with the Declaration of Helsinki and its updates, and the study protocol was approved by the local Ethics Committee (UCAM—TC/05-24).

### 2.2. Design

Participants completed three overground walking trials at a self-selected, comfortable speed along a straight 10-m walkway. All materials and methods were consistent with the laboratory’s recently published standardized gait analysis protocol [27]. As such, the first and last 2 m of the walkway were designated as acceleration and deceleration zones [3], respectively, and the central region was designated as the steady-state walking region [3,27]. Because OptoGait provides measurements only within its instrumented region, between-device agreement analyses were restricted to full strides occurring within the 4-m OptoGait capture area (i.e., the area concurrently measured by both systems).

### 2.3. Instrumentation

#### 2.3.1. 3D Markerless Motion Capture—OpenCap (CM)

A two-camera setup (OpenCap) was used with two iOS devices (series 10 and 12) mounted on tripods at 80 cm and positioned 2 m laterally from the walkway midline (4 m between cameras). Video was recorded at 720 × 1280 pixels and 60 Hz. The OpenCap workflow was used to estimate 2D keypoints in each camera view and triangulate 3D positions to compute gait outcomes. OpenCap initializes an OpenSim-based biomechanical model from a neutral calibration pose with published modifications described in the OpenCap best practices [28].

#### 2.3.2. OptoGait LED System (OPT)

OptoGait (Microgate Srl, Bolzano, Italy) consisted of four 1-m bars, positioned on either side of the walking path to define the 4-m capture area. Data were sampled at 1000 Hz and saved to a PC using OptoGait software (v1.6.4.0; Microgate Srl, Bolzano, Italy). Only steps detected within the OptoGait bars were included in analyses, and data were cleaned post hoc to exclude incomplete steps that were not fully within the 4-m capture area.

### 2.4. Calibration

Camera placement and calibration followed our recently published OpenCap standardized gait analysis protocol [27]. The protocol specifies the 10-m walkway segmentation (2 m acceleration + 6 m measurement + 2 m deceleration), an 80 cm camera height, 2-m lateral placement, and the use of a fixed reference location (floor marker) for consistent calibration-board placement and neutral pose positioning [27]. Following camera calibration, participants completed the CM neutral pose calibration in a relaxed anatomical stance prior to walking trials, as suggested in the OpenCap best practices published online [28]. To maintain capture quality and reduce tracking variability, participant attire and environment were aligned with the standardized protocol recommendations (form-fitting clothing; consistent lighting to minimize shadows and improve key point tracking) [27,28].

### 2.5. Procedures

**Figure 1:**
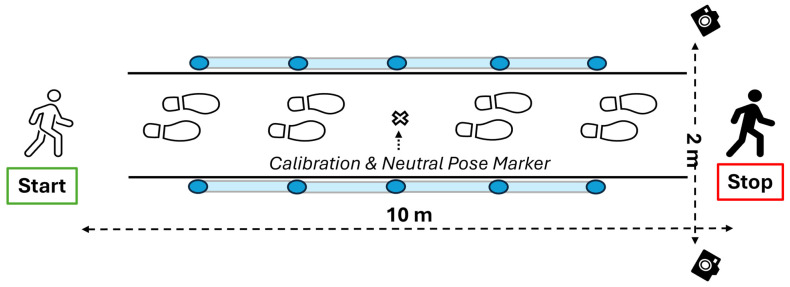
Walking trials experimental setup.

Participants performed three 10-m walking trials at a comfortable walking speed. Instructions were standardized as: “You will complete a series of walking trials at a comfortable walking pace. A ‘comfortable’ walking pace is your everyday walking pace, which you would typically use in your normal activities when not in a rush. The instructions will be “comfortable walking pace, all the way through the white line, Ready, Set, GO! Only start after I say GO.” Between trials, participants waited as needed for CM processing when applicable (OPT processing is near-immediate, whereas CM requires video upload/processing time). Figure 1 illustrates the experimental setup and walking path, showing the positioning of the CM and the OPT system relative to the 10-m walkway.

### 2.6. Data Extraction

Spatiotemporal gait parameters of interest were gait speed (m/s), stride length (m), cadence (steps/min), and double support time (DS; % gait cycle). CM gait parameters were extracted post-collection using OpenCap’s Advanced Overground Gait Analysis, which identifies the last viable right-leg stride and computes all parameters from that stride alone. The automated overground analysis implemented in OpenCap was intentionally used to reflect real-world deployment conditions, where rapid processing and standardized outputs would be prioritized. OPT gait parameters were extracted from OptoGait software from the average of all valid strides within the 4-m capture area. Three consecutive trials per participant, per device, collected under identical conditions without intervening tasks or recalibration, were used to assess within- and between-device reliability by quantifying trial-to-trial consistency. Within-device reliability analyses used all trials per participant. Between-device agreement analyses were performed at the participant level using the mean of all valid trials available per device. When an OpenCap trial failed to process, the device mean was computed using the two remaining valid trials; no participant had more than one failed OpenCap trial.

### 2.7. Statistical Analysis

Data was analyzed using IBM SPSS Statistics for Windows, version 25 (IBM Corp., Armonk, NY, USA). The syntax for SPSS was generated by ChatGPT 5.2 (OpenAI, San Francisco, CA, USA, 2025). Descriptive statistics are presented as mean (M) ± standard deviation (SD) and were computed for each variable. To determine within-device relative reliability, within-session reliability across three trials was quantified separately for each device using a two-way mixed-effects, absolute-agreement ICC model: ICC (3,1) for single trials and ICC (3,3) for the mean of three trials, with 95% CIs derived from the F distribution [29,30]. This model was selected because the analysis focused on the applied repeatability of the specific measurement systems used in this study (OpenCap and OptoGait), which were treated as fixed devices rather than a random sample. Interpretation of the ICC values follows Koo and Li (2016): <0.50 = poor; 0.50–0.75 = moderate; 0.75–0.90 = good; >0.90 = excellent, using the 95% CI range rather than the absolute ICC value [31].

To determine within-device absolute reliability, it was quantified using a one-factor repeated-measures general linear model (GLM) with trial as the within-subject factor. The within-subject mean square error from this model (MSe) was used to compute the within-subject standard deviation as SD (within) = √MSe, which, in the literature, is equivalent to the standard error of measurement (SEM). Meaningful detectable change (MDC) represents the smallest difference in two single-trial measurements that can be interpreted as real (exceeding measurement error). For further explanation and calculations, please refer to our laboratory’s article regarding a standardized gait analysis protocol using markerless motion capture [27]. As is common with standard human gait research, within-session reliability indices reflect the combined effects of biological trial-to-trial gait variability and measurement-related error and therefore quantify the applied repeatability of each system under real-world walking conditions rather than isolated sensor noise. Between-device agreement (OPT vs. CM) was evaluated using participant-level means. Systematic bias was assessed with paired-samples *t*-tests (OPT − CM) and 95% CIs; association was assessed using Pearson correlations (r). Absolute agreement was quantified using ICC (3,1) (two-way mixed, absolute agreement; participants random, devices fixed). Bland–Altman plots were generated in MATLAB (2025; The MathWorks, Inc.; R2025a; Natick, MA) using a script that was generated by ChatGPT 5.2, using the mean-versus difference (OPT − CM) format, with bias and 95% limits of agreement (bias ± 1.96 × SDdiff). The present study is not intended as a sensor-level performance or accuracy evaluation, but rather as an assessment of applied repeatability and practical comparability between two gait assessment tools under standardized human gait conditions.

## 3. Results

### 3.1. Participant Demographics

Thirty-nine participants completed the protocol. Demographic data are presented in Table 1. Demographics for men and women are presented separately to be transparent and to help understand and apply the results regarding walking patterns, since body measurements that often differ between men and women can affect gait. Demographic statistics were for descriptive purposes and were not the primary focus of the study.

### 3.2. Within-Device Reliability

In total, 117 walking trials were performed (39 participants × 3 trials), and 111 trials (94.9%) were successfully processed using the OpenCap overground gait analysis program. Six trials (5.1%) failed to process in the overground gait analysis. All 117 trials from the OPT system were processed and utilized. Therefore, within-device reliability is based on an *n* = 33 for the CM system and *N* = 39 for the OPT system.

For gait speed, stride length, and cadence, both systems demonstrated good-to-excellent reliability for single trials and excellent reliability for the mean of three trials. Specifically, OPT ICC (3,1) values were 0.871 (speed), 0.734 (stride length), and 0.920 (cadence), with ICC (3,3) values ranging from 0.953 to 0.972. CM ICC (3,1) values were 0.835 (speed), 0.791 (stride), and 0.917 (cadence), with ICC (3,3) values ranging from 0.919 to 0.971. Double support showed lower relative reliability than the other outcomes (OPT ICC (3,1) = 0.527; CM ICC (3,1) = 0.647). Within-device ICCs are reported in Table 2.

For gait speed, stride length, and cadence, SEM (1) and MDC (1) were small relative to the grand means for both systems and improved when averaging three trials. For example, OPT SEM (1) was 0.053 m/s for gait speed and decreased to SEM (3) = 0.032 m/s; CM SEM (1) was 0.064 m/s and decreased to SEM (3) = 0.039 m/s. Similar reductions were observed for stride length and cadence. Double support exhibited the largest error, with OPT SEM (1) = 3.226% GC and MDC (1) = 8.943% GC (reduced to SEM (3) = 2.250% GC; MDC (3) = 6.236% GC), while CM showed lower DS error than OPT. Absolute reliability indices are presented in Table 3.

### 3.3. Between-Device Agreement (OPT vs. CM)

Between-device agreement analyses were conducted at the participant level (*n* = 39) using each device’s mean across all valid trials. For participants with an OpenCap processing failure, the OpenCap mean was calculated from the remaining two valid trials; no participant had more than one failed OpenCap trial, and only 6 participants were affected.

CM produced higher mean values than OPT for gait speed (1.412 ± 0.159 vs. 1.302 ± 0.142 m/s), stride length (1.554 ± 0.118 vs. 1.427 ± 0.093 m), and DS (28.71 ± 2.81 vs. 25.54 ± 3.89% GC). Cadence values from both devices were highly similar (CM: 108.33 ± 7.49 spm; OPT: 108.92 ± 7.55 spm). Paired comparisons showed significant systematic bias for all outcomes: gait speed bias = −0.110 m/s (95% CI −0.126 to −0.094; *p* < 0.001), stride length bias = −0.127 m (95% CI −0.146 to −0.107; *p* < 0.001), cadence bias = 0.59 spm (95% CI 0.13 to 1.04; *p* = 0.013), and DS bias = −3.17% GC (95% CI −4.39 to −1.95; *p* < 0.001). Pearson correlations between devices were high for gait speed (r = 0.951), stride length (r = 0.864), and cadence (r = 0.983), but moderate for DS (r = 0.405). Between-device descriptive values and paired comparisons are reported in Table 4.

Between-device ICC (3,1) absolute agreement values are presented in Table 5. Cadence showed excellent agreement (ICC = 0.980; 95% CI 0.958–0.990), gait speed was lower with a wide CI (ICC = 0.748; 95% CI −0.060–0.932), stride length was lower (ICC = 0.493; 95% CI −0.080–0.816), and DS was the lowest (ICC = 0.271; 95% CI −0.048–0.543).

**Figure 2:**
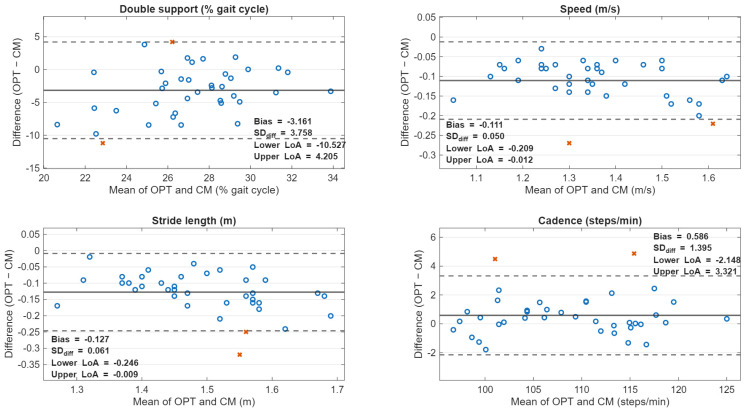
Bland–Altman Plots: OptoGait to OpenCap (OPT-CM).

Bland–Altman plots are shown in Figure 2. Limits of agreement (OPT − CM) were approximately −10.55 to 4.21% GC for DS, −0.208 to −0.012 m/s for gait speed, −0.245 to −0.009 m for stride length, and −2.15 to 3.32 spm for cadence.

Bland–Altman plots illustrating agreement between the OptoGait walkway (OPT) and the OpenCap 3D markerless system (CM) for (A) double support (% gait cycle), (B) gait speed (m/s), (C) stride length (m), and (D) cadence (steps/min). For each outcome, the *x*-axis shows the mean of OPT and CM, and the *y*-axis shows the difference between systems (OPT − CM). The solid horizontal line represents the mean difference (bias), and the dashed lines represent the 95% limits of agreement (bias ± 1.96 × SD of the differences). Points falling outside the limits of agreement are red x’s and indicate participants for whom device disagreement exceeded the expected measurement error. OPT = OptoGait; CM = OpenCap; DS = double support.

## 4. Discussion

The purpose of this study was to evaluate the concurrent validity and within-session reliability of OpenCap-derived spatiotemporal gait parameters relative to an established photoelectric cell system (OptoGait) in young, healthy adults. Under a standardized laboratory setup, both systems demonstrated good-to-excellent within-session reliability for gait speed, stride length, and cadence, whereas double support (DS) showed lower reliability and larger absolute errors.

Within-device relative reliability was strong for the primary outcomes. For single-trial estimates, OPT and CM demonstrated good-to-excellent ICC (3,1) values for gait speed (OPT: 0.871; CM: 0.835), stride length (OPT: 0.734; CM: 0.791), and cadence (OPT: 0.920; CM: 0.917), with excellent reliability when averaging three trials (ICC (3,3) ≈ 0.89–0.97). For context, other gait measurement technologies, including photoelectric walkways, marker-based motion capture, wearable sensors, and more recent markerless video systems, have consistently demonstrated good to excellent within-session reliability for key spatiotemporal variables such as gait speed, stride length, and cadence, with ICC values ranging from 0.78 to 0.98 [17,21,32,33]. Therefore, these findings support the use of either system for within-session quantification of these spatiotemporal outcomes in healthy gait, particularly when averaging multiple trials is possible.

Absolute reliability indices further contextualize the magnitude of trial-to-trial noise. For CM, SEM (1) values were small for gait speed (0.064 m/s), stride length (0.052 m), and cadence (2.220 spm), with corresponding MDC (1) values of 0.178 m/s, 0.144 m, and 6.152 spm, respectively; these improved with three-trial averaging (e.g., MDC (3) for gait speed = 0.109 m/s). A similar pattern was observed for OPT, with slightly smaller MDC (1) for gait speed (0.147 m/s) but broadly comparable precision across outcomes. In contrast, DS exhibited the largest error for both systems, particularly in OPT (SEM (1) = 3.226% GC; MDC (1) = 8.943% GC), reinforcing that temporal phase metrics are more sensitive to event-detection differences and trial-to-trial fluctuations than pace/rhythm gait parameters. Double-support specifically relies on accurately determining when heel strike and toe off occur, which occur within a small temporal window. This makes it easy for small tracking mistakes to affect the overall results. In markerless motion capture systems, these moments are inferred from body motion rather than measured directly, which can lead to timing errors that are worse than those in spatiotemporal parameters such as gait speed or stride length.

The reliability framework used in this study follows the same approach as that used to evaluate and adopt clinical spatiotemporal gait systems, such as photoelectric walkways. In these cases, repeatability across repeated human walking trials is the basis for judging measurement precision and meaningful change. In this context, separating biological variability from system error is neither practical nor necessary for establishing reliable measurements. Therefore, from a practical standpoint, these MDC values indicate the minimum change that must be exceeded to interpret within-session differences as “real” (beyond measurement error). For example, in many clinical contexts, small meaningful changes in comfortable gait speed have been estimated at 0.05 m/s, with substantial change closer to 0.10 m/s [8,34]. In the current research, CM single-trial MDC for gait speed (0.178 m/s) exceeds these commonly used anchors, whereas the three-trial average MDC (0.109 m/s) approaches the “substantial change” range. This further supports collecting multiple trials when CM is used to detect modest changes (e.g., due to an intervention) within a session. In our recently published protocol paper, we averaged five trials, and our MDC across the averaged trials was 0.91 m/s, which falls within the commonly used anchors in the literature [27].

The current results show that OpenCap can be a valuable tool for measuring spatiotemporal gait, similar to established photoelectric systems. It is important to interpret measurements within the device’s precision limits and to use them consistently in each study or clinical process. Doing so helps ensure that data collected with OpenCap are reliable and valid in both research and clinical settings.

Despite strong associations between systems for gait speed, stride length, and cadence (r = 0.864–0.983), CM produced systematically higher mean values than OPT for gait speed (+0.110 m/s), stride length (+0.127 m), and DS (+3.17% GC), with a smaller but statistically significant cadence difference (−0.59 spm). As expected, these systematic offsets reduced absolute agreement ICCs, particularly for stride length and DS (ICC (3,1) = 0.493 and 0.271, respectively), even when correlations were high. In other words, the two systems tracked inter-individual differences similarly (high r), but they were not directly interchangeable without accounting for bias.

Bland–Altman limits of agreement provide added information regarding the magnitude of device disagreement at the individual level. The limits were relatively narrow for cadence (≈−2.15 to 3.32 spm) but broader for DS (≈−10.55 to 4.21% GC), and the speed/stride length limits were largely negative (reflecting OpenCap’s higher values). These results suggest that CM may be suitable as a standalone tool (given its within-device reliability), but mixing CM and OPT values within the same longitudinal dataset or comparing outcomes across studies using different systems could confound interpretation unless a correction strategy is applied (e.g., device-specific reference values, regression-based conversion, or consistent use of a single device per participant/timepoint).

Several methodological differences likely contributed to the observed biases. First, OpenCap’s Advanced Overground Gait Analysis computes spatiotemporal parameters from the last viable right-leg stride only, whereas OPT derives parameters from the average of all valid steps within the capture area. A single-stride estimate is inherently more sensitive to stride-to-stride fluctuations and to where, spatially, that stride occurs within the walkway (even with acceleration/deceleration zones). Conversely, averaging across multiple steps/strides tends to dampen variability and may better reflect steady-state behavior across the instrumented area. We attempted to limit this difference by including only full strides in the capture area via post hoc step elimination in the OptoGait software; however, it is more challenging to identify and compare the exact strides between the OpenCap and the OptoGait systems. Second, the systems differ fundamentally in how they determine gait events. OPT detects foot contact through interruptions of photoelectric beams sampled at high frequency (1000 Hz). It detects foot contact by interrupting slightly elevated photoelectric beams (approximately 3 mm off the ground). It can register initial contact earlier and toe-off later. In contrast, CM relies on video (60 Hz) plus pose estimation, 3D reconstruction, and event inference within its processing pipeline. These differences are expected to disproportionately affect DS and other phase-based measures that depend on the precise timing of initial contact and toe-off [35,36,37]. The comparatively weaker between-device association for DS (r = 0.405) and the lower absolute agreement ICC support this interpretation.

Finally, even when both systems are used simultaneously, they may not quantify the same stride(s) within the shared capture region. Although between-device analyses were restricted to the OPT capture area, CM “last viable stride” selection may not always align with the exact strides OPT includes, especially if tracking confidence or segmentation rules differ across frames. Future work would benefit from software workflows that compute outcomes from multiple consecutive strides and allow explicit spatial-temporal alignment of the analyzed steps across devices.

A key aim of this manuscript is to situate the present CM reliability/precision estimates within the context of the lab’s recently published standardized protocol paper [27]. That study reported CM absolute reliability values during comfortable walking that are closely aligned with the present results: SEM (1) for gait speed (0.074 m/s), stride length (0.059 m), cadence (2.81 spm), and DS (2.03% GC), with corresponding MDC (1) values of 0.205 m/s, 0.163 m, 7.790 spm, and 5.636% GC. In the current study, CM SEM (1) and MDC (1) were slightly smaller for gait speed (0.064 m/s; 0.178 m/s), stride length (0.052 m; 0.144 m), and cadence (2.220 spm; 6.152 spm), and comparable for DS (1.970% GC; 5.461% GC).

Two methodological considerations help interpret these slight differences. First, the standardized protocol paper primarily emphasized the mean of five trials (MDC (5) for speed = 0.091 m/s), whereas the present study used a three-trial average (MDC (3) for OpenCap speed = 0.109 m/s). This is consistent with classical measurement theory: averaging more trials reduces the SEM/MDC approximately in proportion to 1/√n, so slightly larger averaged-trial MDC values are expected when *n* = 3 rather than *n* = 5. Second, both studies examined young, healthy adults walking under controlled, standardized camera placement and calibration conditions; thus, the close agreement in SEM/MDC values across papers supports the reproducibility of OpenCap’s within-session precision when a standardized protocol is followed. Therefore, under standardized laboratory conditions in young healthy adults, CM demonstrated within-session reliability and measurement precision comparable to OPT for gait speed, stride length, and cadence, supporting its use as a practical alternative for spatiotemporal gait assessment when a single system is used consistently.

The present findings are consistent with the broader markerless gait analysis literature, which shows that spatiotemporal measures such as speed, cadence, and step/stride length typically achieve higher reliability than measures that rely on finer event timing or frontal-plane estimation [21]. They are also compatible with prior work, indicating that OPT can provide a reliable spatiotemporal assessment in healthy gait, though phase-based measures can be more sensitive to methodological factors and detection rules [17]. In this context, the main contribution of the current paper is not to argue that one system is universally “better,” but to quantify the within-device consistency of each system under matched conditions and the extent and direction of systematic differences when the tools are compared directly. Despite strong associations between devices, systematic bias and limited absolute agreement for stride length and double support indicate that CM and OPT should not be considered interchangeable without device-specific correction. However, cadence exhibited excellent agreement and may be comparable across systems. It should be noted that the current research sought to contrast spatiotemporal measures from the CM device with those from the established OPT device, and the authors believe it has done just that; however, it cannot be overstated that the two systems should not be compared or considered interchangeable. Therefore, the authors believe that the observed agreement and reliability in this study should be interpreted as indicators of applied repeatability under standardized overground walking conditions. These results do not provide evidence of measurement equivalence or intrinsic system accuracy. It is important for future studies to further investigate these aspects using more rigorous reference standards.

Several limitations should be noted. First, as is typical in initial validation studies, this work focused on healthy adults to establish baseline reliability and measurement error under controlled conditions, a necessary prerequisite for extending analyses to populations with atypical kinematics or higher gait variability, which can challenge pose tracking and event inference. Future work should examine the performance of markerless systems in clinical populations with altered gait mechanics. Second, reliability was assessed within-session; between-day test–retest reliability (including re-setup and recalibration) remains important for clinical and longitudinal applications. Third, OpenCap’s current overground workflow relies on a single selected stride, which may not fully represent trial-level gait behavior and complicates stride-level matching to instrumented walkways. Future work should evaluate multi-stride aggregation approaches in OpenCap, examine whether device-specific biases remain stable across speeds and conditions, and determine whether simple calibration equations can improve absolute agreement sufficiently to allow cross-device comparisons for specific outcomes (particularly speed and stride length). Fourth, the current study did not separate system-specific measurement error from the biological variability observed between repeated gait trials, as all spatiotemporal parameters were obtained from multiple human walking trials rather than repeated measures of the same motion. As a result, the reliability data presented here reflect the repeatability achievable under real-world conditions rather than the sensor’s intrinsic precision. Lastly, the current research did not use a gold-standard criterion measure to validate OpenCap. Future research using marker-based motion capture simultaneously could help identify and reduce errors inherent to the system, thereby making measurements more reliable. However, it is important to remember that these methods should be used alongside, not instead of, the practical methods we use to assess reliability needed for real-world walking tests. Both methods are needed to advance the field forward and ensure the data are both scientifically relevant and useful in practice.

## 5. Conclusions

Under standardized laboratory conditions, CM produced consistent spatiotemporal gait estimates and demonstrated reliability comparable to that of an established photoelectric walkway system, supporting its use as a practical, easily deployable option for clinical and field-based gait assessment when instrumented systems are not feasible. At the same time, systematic differences between CM and OPT indicate that the two systems should not be considered universally interchangeable, particularly for parameters that depend strongly on precise gait event timing. Accordingly, CM is best positioned as a standalone measurement tool, applied consistently within a given study or clinical pathway, with interpr31etation of change guided by device-specific measurement error thresholds rather than direct comparison with values from other systems. These findings are comparable to our recently published standardized gait analysis using 3D markerless motion capture, reinforcing the reproducibility achievable with standardized setup procedures and multiple trials and highlighting OpenCap’s potential to expand access to quantitative gait assessment beyond traditional laboratory environments.

## Figures and Tables

**Table 1:** Participant baseline demographics—presented as mean ± standard deviation.

	Total (*N* = 39)	Female (*n* = 15)	Male (*n* = 24)	*p*	*g*
Age (years)	24.14 ± 5.3	22.67 ± 2.13	25.06 ± 6.47	0.104	0.454
Height (cm)	175.36 ± 9.36	169.17 ± 10.22	179.23 ± 6.37	**0.003**	1.25
Weight (kg)	74.49 ± 13.69	67.22 ± 16.82	79.04 ± 9.02	**0.007**	0.941
BMI	24.17 ± 3.67	23.38 ± 4.43	24.67 ± 3.11	0.290	0.352

*p* = *p*-value, *g* = Hedges *g* effect size. *p*-Values reflect independent-samples comparisons (two-tailed) between female and male participants for descriptive purposes only. **Bold** indicates statistical significance.

**Table 2:** Intraclass Correlation Coefficients for within-subject test–retest.

Variable	Device	ICC (3,1) [95% CI]	Interpretation (3,1)	ICC (3,3) [95% CI]	Interpretation (3,3)
Gait Speed (m/s)	OPT	0.871 [0.788–0.926]	Good-Excellent	0.953 [0.918–0.975]	Excellent
	CM	0.835 [0.723–0.912]	Moderate-Excellent	0.938 [0.889–0.966]	Good-Excellent
Stride Length (m)	OPT	0.734 [0.582–0.842]	Moderate-Good	0.892 [0.813–0.939]	Good-Excellent
	CM	0.791 [0.655–0.884]	Moderate-Good	0.919 [0.857–0.955]	Good-Excellent
Cadence (spm)	OPT	0.920 [0.867–0.954]	Good-Excellent	0.972 [0.954–0.983]	Excellent
	CM	0.917 [0.863–0.952]	Good-Excellent	0.971 [0.952–0.982]	Excellent
DS (% of gait cycle)	OPT	0.527 [0.320–0.694]	Poor-Moderate	0.770 [0.613–0.874]	Moderate-Good
	CM	0.647 [0.449–0.795]	Poor-Moderate	0.846 [0.689–0.924]	Moderate-Excellent

ICC = Intraclass correlation coefficient; values derived from a two-way mixed-effects, absolute-agreement model. ICC (3,1) represents reliability for a single trial; ICC (3,3) represents reliability for the mean of three trials. Interpretation follows Koo and Li (2016) [31]: <0.50 = poor; 0.50–0.75 = moderate; 0.75–0.90 = good; >0.90 = excellent using the 95% CI range rather than the absolute ICC value. OPT (*N* = 39) = OptoGait; CM (*n* = 33) = OpenCap.

**Table 3:** Absolute reliability of spatiotemporal gait variables.

Variable	Device	Mean	SD (Pooled)	SD (Within)	SEM (1)	MDC (1)	SEM (3)	MDC (3)
Gait Speed (m/s)	OPT	1.302	0.148	0.055	0.053	0.147	0.032	0.089
	CM	1.412	0.158	0.063	0.064	0.178	0.039	0.109
Stride Length (m)	OPT	1.427	0.102	0.055	0.052	0.145	0.033	0.093
	CM	1.540	0.114	0.055	0.052	0.144	0.032	0.090
Cadence (spm)	OPT	108.92	7.748	2.123	2.192	6.075	1.297	3.594
	CM	108.86	7.704	2.111	2.220	6.152	1.312	3.637
DS (% gait cycle)	OPT	25.54	4.691	3.194	3.226	8.943	2.250	6.236
	CM	28.582	3.316	1.981	1.970	5.461	1.301	3.607

Mean represents the average of all trials. SD (pooled) = standard deviation of all individual trials across subjects and repetitions. SD (within) = √MS_e_, where MS_e_ is the mean-square error term from the within-subjects effect of the repeated-measures GLM with trial as the within-subject factor. SEM (1) = standard error of measurement in the original units. MDC (1) = minimal detectable change between two single-trial measurements at the 95% confidence level. SEM (3) and MDC (3) are the corresponding indices for the average of three trials. OPT (*N* = 39) = OptoGait; CM (*n* = 33) = OpenCap.

**Table 4:** Agreement between OptoGait and OpenCap for spatiotemporal gait variables.

Variable	Device	Mean	SD	Bias (OPT-CM) [95% CI]	*r*	*t*(38)	*p*	*d*
Gait Speed (m/s)	OPT	1.302	0.142	−0.110 [−0.126, −0.094]	0.951	−13.80	<0.001	0.730
	CM	1.412	0.159					
Stride Length (m)	OPT	1.427	0.093	−0.127 [−0.146, −0.107]	0.864	−13.18	<0.001	1.20
	CM	1.554	0.118					
Cadence (spm)	OPT	108.92	7.55	0.59 [0.13, 1.04]	0.983	2.62	0.013	0.078
	CM	108.33	7.49					
DS (% gait cycle)	OPT	25.54	3.89	−3.17 [−4.39, −1.95]	0.405	−5.25	<0.001	0.934
	CM	28.71	2.81					

Values are presented as Means and SD across participants (*n* = 39). OPT = OptoGait; CM = OpenCap (camera-based). “OPT–CM” represents the paired mean difference (bias) computed as OPT minus CM. *r* = the Pearson correlation between devices. *t*(38) and *p* values are derived from two-tailed paired-samples *t*-tests. Participant outcomes reflect the mean of trials per device.

**Table 5:** Intraclass Correlation Coefficients for absolute agreement.

Variable	ICC (3,1) [95% CI]	Interpretation
Gait Speed (m/s)	0.748 [−0.060, 0.932]	Poor-Excellent
Stride Length (m)	0.493 [−0.080, 0.816]	Poor-Good
Cadence (spm)	0.980 [0.958, 0.990]	Excellent
DS (% of gait cycle)	0.271 [−0.048, 0.543]	Poor-Moderate

ICCs reflect a two-way mixed-effects model with absolute agreement (ICC (3,1) participants random, devices fixed). Each device value represents the participant’s mean of trials.

## Data Availability

Data are available upon reasonable request by contacting the corresponding author.

## Abbreviations

The following abbreviations are used in this manuscript:

3D	Three-dimensional
MMC	Markerless motion capture
CM	OpenCap
DS	Double support
ICC	Intra-class correlation
LoA	Limitation of Agreement
MSe	Mean square error
OPT	OptoGait
SEM	Standard error of measurement
MDC	Minimal detectable change
